# A Synchronized Multipoint Vision-Based System for Displacement Measurement of Civil Infrastructures

**DOI:** 10.1100/2012/519146

**Published:** 2012-09-17

**Authors:** Hoai-Nam Ho, Jong-Han Lee, Young-Soo Park, Jong-Jae Lee

**Affiliations:** ^1^Department of Civil and Environmental Engineering, Sejong University, Seoul 143-747, Republic of Korea; ^2^Research and Engineering Division, POSCO E&C, Incheon 406-732, Republic of Korea

## Abstract

This study presents an advanced multipoint vision-based system for dynamic displacement measurement of civil infrastructures. The proposed system consists of commercial camcorders, frame grabbers, low-cost PCs, and a wireless LAN access point. The images of target panels attached to a structure are captured by camcorders and streamed into the PC via frame grabbers. Then the displacements of targets are calculated using image processing techniques with premeasured calibration parameters. This system can simultaneously support two camcorders at the subsystem level for dynamic real-time displacement measurement. The data of each subsystem including system time are wirelessly transferred from the subsystem PCs to master PC and vice versa. Furthermore, synchronization process is implemented to ensure the time synchronization between the master PC and subsystem PCs. Several shaking table tests were conducted to verify the effectiveness of the proposed system, and the results showed very good agreement with those from a conventional sensor with an error of less than 2%.

## 1. Introduction

Large-scale civil structures including bridges and buildings are exposed to various loads such as traffic loads and/or natural disasters (e.g., earthquakes, typhoons, cyclones, blizzards). Monitoring structural displacement under such dynamic loads plays an essential role in structural health monitoring. In fact, the direct measurement of structural displacement responses has been a challenge, especially for large-scale structures because traditional sensors such as the linear variable transformer (LVDT) require a stationary reference that is difficult to find a proper location in the field. In recent years, the global position system (GPS) [1–3] and the laser doppler vibrometer [4] have emerged as new noncontact measurement techniques, but their applications are still limited as a result of their high cost.

With increases in CPU capabilities, improvements in image capturing devices and the development of new postprocessing image algorithms, vision-based displacement measurement is becoming one of the most common noncontact measurement techniques in civil engineering applications [5–9]. Compared with the other sensors, vision-based measurement provides several advantages: (1) it can provide direct measurements in both the time and the three-dimensional (3D) displacement; (2) it can measure displacement at multiple locations simultaneously in cost-effective manner; (3) it needs a less complicated and labor intensive setup.

Various vision-based systems that measure structural displacement have been developed. Wahbeh et al. [5] developed a high-fidelity video camera with a resolution of 520 lines and a digital zoom of 450 capabilities. The targets consisted of a 28 × 32 inch black steel sheet on which two high-resolution LEDs were mounted to measure the displacement of the Vincent Thomas Bridge, located in San Pedro, California. Then, vision-based systems for measuring the dynamic displacement of bridges in real time was introduced by the authors [6, 7] in 2006 and 2007, respectively. They attached target panels to a structure, captured moving targets by camcorders, and then calculated the amount of structural displacement by applying image processing techniques. In 2009, Fukuda et al. [8] presented a cost-effective (a term previously coined by Lee and Shinozuka [6]) vision-based displacement measurement applied to large-size civil engineering structures, such as bridges and buildings. They employed a TCP/IP (transmission control protocol/Internet protocol) for communications and carried out time synchronization for the time synchronization of the system. More recently, in 2011, Choi et al. [9] introduced a vision-based structural dynamic displacement system using an economical hand-held digital camera. A recorded video containing dynamic information of target panel attached directly to the civil structure was processed with image resizing method, and mm/pixel coefficient updating process, then the structure displacement was successfully determined by calculating the target position in each frame.

The existing vision-based systems for displacement measurement applied to civil engineering applications still have several limitations. There is little cost-effective system which can measure displacement at multiple locations simultaneously. The cost of frame grabbers which can support a few image capturing devices is still very expensive. Furthermore, it is not easy to increase the number of measurement locations more than the frame grabbers' capacity (usually less than 4). When using multiple frame grabbers, time synchronization for frame grabbers and high speed data transmission can be critical issues to be resolved.

The objective of this study is to introduce a synchronized multipoint vision-based system for dynamic real-time displacement measurement. Because of the huge amount of image data, real-time processing is an important issue to be resolved, particularly in the multipoint vision based system. Compared to the previous vision-based displacement measurement systems, the proposed system provides the following advantages: (1) it provides cost-effective multipoint measurement capabilities (i.e., it can support two camcorders at the subsystem level and utilize the commercial camcorders for real-time displacement measurement); (2) it allows an increase in the number of measurement points in cost-effective manner; (3) it provides a user-friendly software interface. Furthermore, it uses TCP/IP to transfer the time synchronization and data connection of each subsystem via a network. To verify the efficiency, stability, and accuracy of this system, we conducted several shaking table tests.

## 2. Development of Advanced Vision-Based System

**Figure 1:**
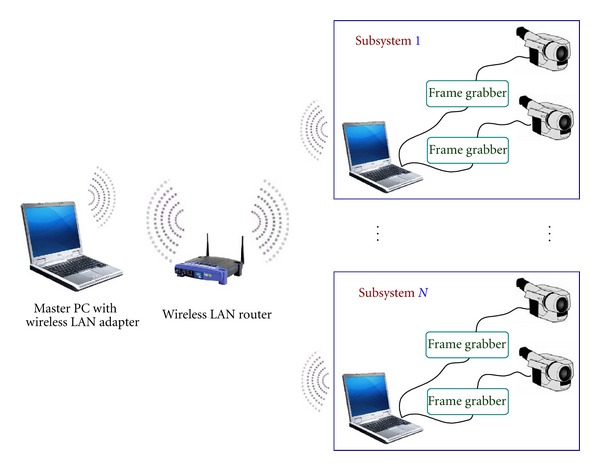
Schematic of the system.

The schematic of the advanced vision-based system for real-time displacement measurement is shown in Figure 1. To measure structural displacement, target panels are attached to a desired location on the structure, and the images of the target panels are captured by camcorders at a remote distance referred to as a “fixed reference point” (the target panels can be marked directly on the structure without using the panels). Then images captured by camcorders are streamed into the slave PC via the frame grabber, and the displacement of the target is calculated by applying image processing techniques with premeasured calibration parameters [7].

### 2.1. Hardware

The hardware is composed of PCs, camcorders (including telescopic lenses that capture better image data from long distances), a wireless LAN router, and frame grabbers. In this system, the PCs can be commercial laptop computers with a minimum 2 GB of RAM. For camcorders, many kinds of available current commercial products can work well with this system. The wireless LAN router should comply with at least an 802.11 g wireless standard to ensure the stability of the data transaction between the master PC and the slave PCs. 

One hardware component that is particularly important is the frame grabber, which is basically considered as a *bridge* that connects the camcorders and the PCs. The frame grabbers help the PCs to adapt to the huge data flow from the single or multiple external camcorders. Subsequently, a low-quality frame grabber will result in poor performance and instability of the entire system. For our system, one commercial frame grabber called *myVision USB *[10] is selected based on the following factors.

- It is easy to move and connect, because the subsystems are designed to work outdoors with laptop PCs, the frame grabber has to be portable, small, and light weight.
- It supports a minimum frame size of 640 × 480 pixels.
- It has low power consumption.
- It provides adequate frame quality at a reasonable cost.

### 2.2. Software

**Figure 2:**
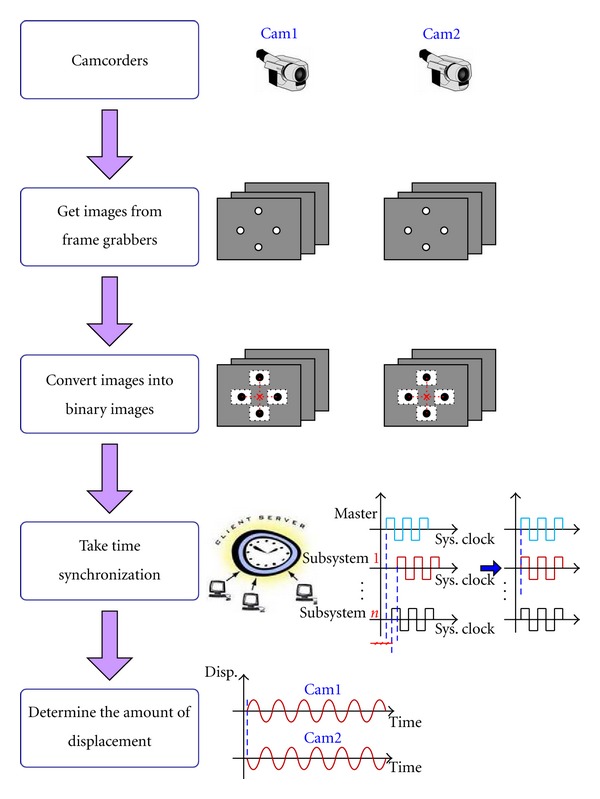
Flowchart of the software.

The software consists of two subprograms implemented by Visual C++ language. One is for the master PC and the other for the slave PCs. The general flowchart of the software program is depicted in Figure 2. Image signals are transferred from camcorders to frame grabbers, and then the real-time images are formed through implementation of the DiretShow libraries of the software. Since the start time of the camcorders can differ depending on the camcorder vendor and type, this system used frame gabbers to initialize the start time and send the frames immediately when required without waiting for the camcorders to start capturing. This connection method, which requires very little time for resetting up the system, can change camcorders that are out of order easily and conveniently, upgrade new camcorder models, and so on without changing or modifying the software. 

**Figure 3:**
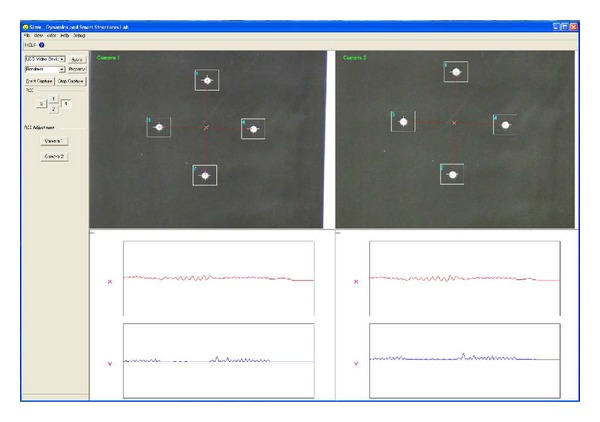
Slave program.

**Figure 4:**
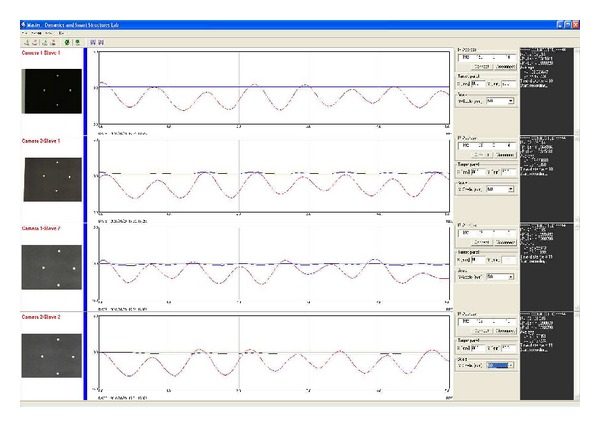
Master program.

The working window of the slave and master programs are shown in Figures 3 and 4, respectively. Initially, each region of interest (ROI) must be selected manually by clicking on the white dots of the target panels on the screen of the slave PCs. As shown in Figure 3, four ROIs should be defined in each slave screen of a subsystem. Theoretically, we can increase the number of cameras connected to the PCs, but because of computational restrictions the speed of current commercial PCs is not powerful enough to handle more than two camcorders with real-time processing. In addition, before measurements are taken, the IP address and the size of target panels must be accurately defined. 

The master program exhibits variations in the wave forms of the target images of each subsystem defined in the slave program.  Figure 4 exhibits the full-scale images (640 × 480) of two camcorders and the wave forms of horizontal (*X*) and vertical (*Y*) displacements in red and blue lines, respectively. 

#### 2.2.1. Conversion of Images into Binary Images

**Figure 5:**
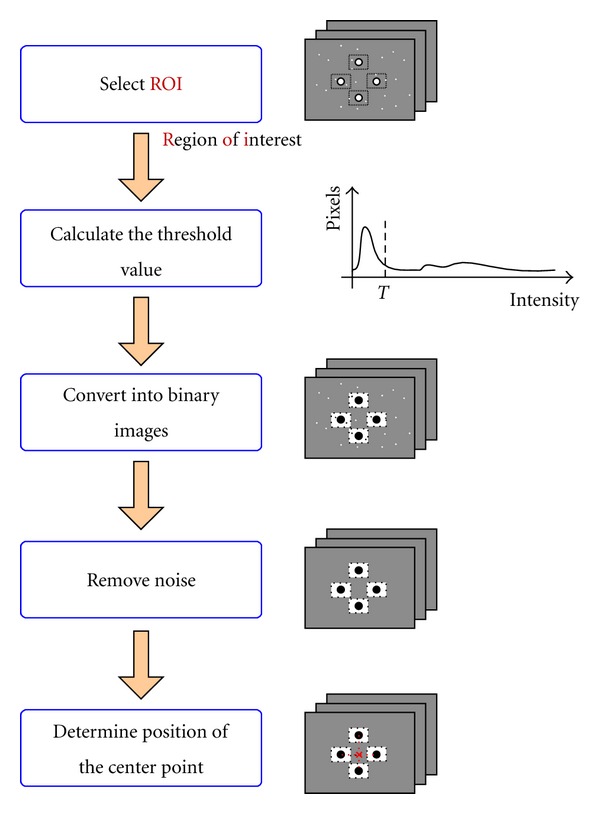
Flowchart of binary image conversion.

**Figure 6:**
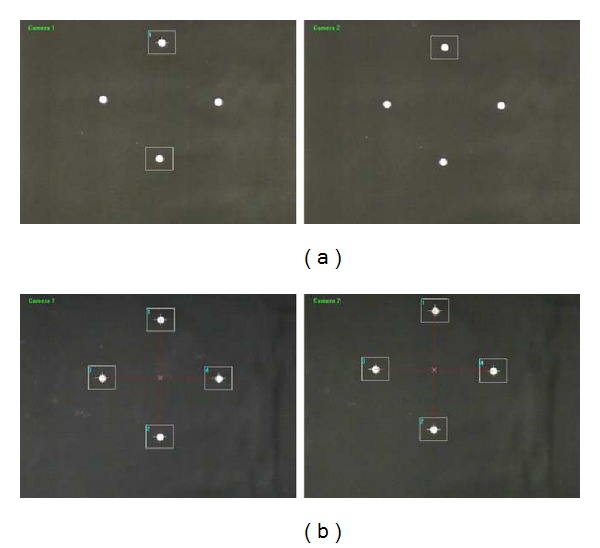
Target recognition. (a) The same threshold value for four spots without adaptive threshold technique and (b) each threshold value for each spot using adaptive threshold technique.

**Figure 7:**
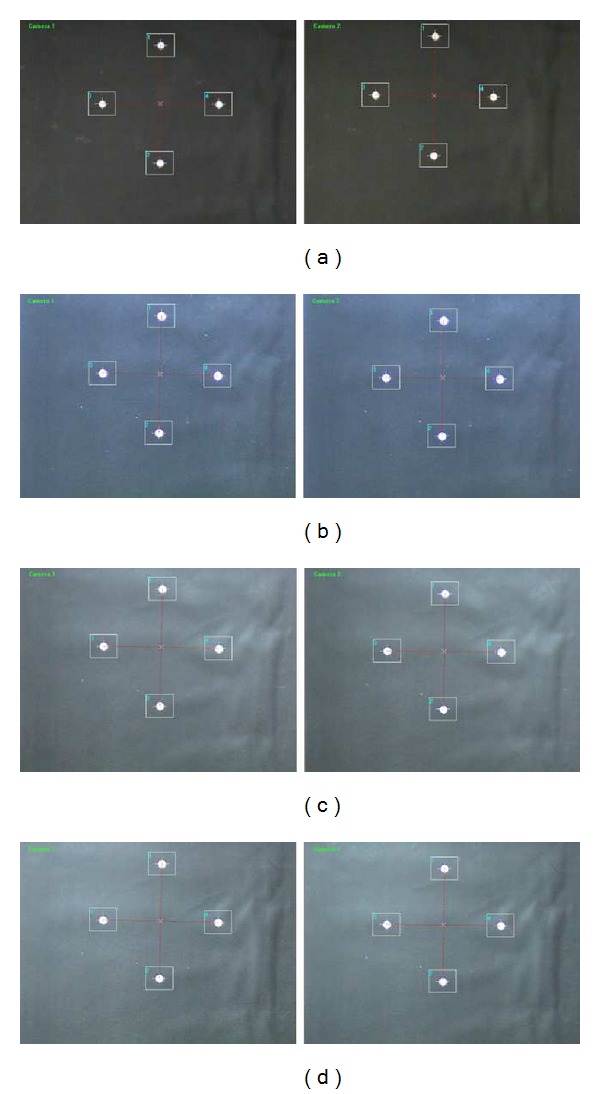
Target recognition in various situations with adaptive threshold technique: (a) dark condition, (b) bright light, (c) bright light in the upper right corner, and (d) bright light in the upper left corner.

To convert grayscale images into binary images, we should begin by determining a suitable threshold value that concisely recognizes the position of target points. Therefore, an adaptive threshold technique using Otsu's method [11] is implemented in this system. The process of binary image conversion using the adaptive threshold value is summarized in Figure 5.  Figure 6 illustrates the significant difference between applying the adaptive threshold algorithm and not applying it. As shown in Figure 6(a), some target points (white spots) can be missed when using an unreasonable threshold value, whereas there is little chance of missing targets when deploying the adaptive threshold algorithm, shown in Figure 6(b).  Figure 7 shows some typical target panel recognitions in different situations using adaptive threshold technique.

#### 2.2.2. System Calibration for Time Synchronization

**Figure 8:**
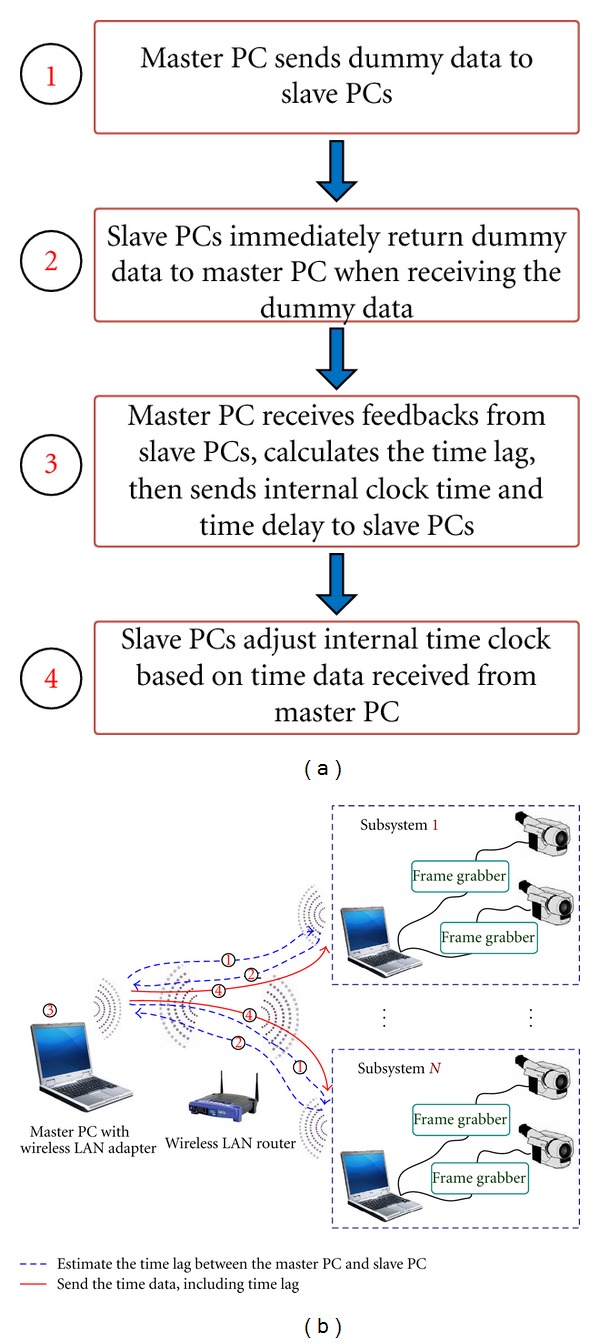
Time synchronization process: (a) time synchronization algorithm and (b) diagram of time synchronization process.

Time calibration process needs to be carried out to maintain time synchronization between the master PC and the two slave PCs, and this important process is clearly shown in Figure 8. The master PC measures the time lag due to the wireless communication and differences in internal time clocks between the master and slave PCs. The master PC first sends the same size of dummy time data to all the slave PCs. When the data reach the slave PCs, they immediately return the received dummy data to the master PC. Then the master PC measures the time gap between the sending and receiving time to calculate the time delay between all the subsystems. Subsequently, the master PC sends the internal clock time and the time delay of the slave PCs to each subsystem. Finally, the slave PCs adjust the internal clock according to the time data received from the master PC. Time is calibrated every 60 s, so the synchronization of time between the master PC and slave PCs remains consistent.

The actual structural displacement can be calculated using trigonometric transformation matrix and scaling factors. The transformation matrix is calculated from the positions of the detected white spots on the target and scaling factors relate the pixel information to the actual geometric information based on the predetermined geometry of the target. More details were explained by Lee et al. [7].

**Figure 9:**
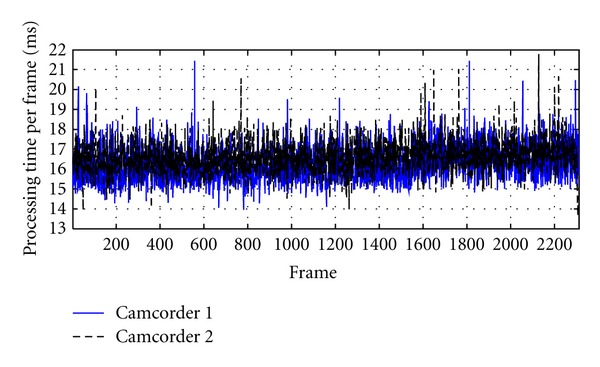
Processing time per frame.

The proposed vision-based system can support two cameras at the subsystem level with 30 frames per second (fps), so it can be appropriate to track the motion of civil structures with the maximum frequency of less than 15 Hz (Nyquist frequency). In reality, civil structures usually have a natural frequency of lower than 4-5 Hz [8], thus the proposed system can be applicable to measuring the dynamic displacement of large-scale structures. However, for the purpose of real-time processing, the processing time per frame should be less than 33.3 milliseconds (ms). To verify the performance of the proposed system, processing time per frame at a subsystem PC was checked as shown in Figure 9. A total of 2300 frames were captured by camcorders and processed at the subsystem. The averaged and maximum processing time per frame are 17 ms and 21.5 ms, respectively, which is fast enough for real-time processing.

## 3. Experimental Verifications

Several laboratory tests were conducted to verify the proposed multipoint vision-based system and the time synchronization algorithm. To ensure good data transaction between the slave subsystems and the master PC, all the PCs and their components should be stable without viruses and spywares before conducting the verification tests. 

### 3.1. Shaking Table Test

**Figure 10:**
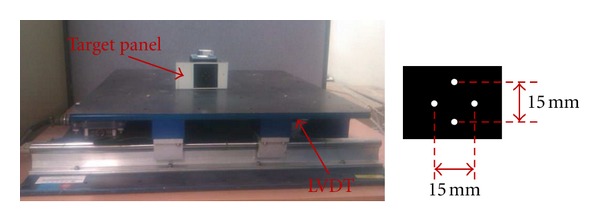
Shaking table and target size.

In order to evaluate the performance and the stability of the proposed synchronized multipoint vision-based system, several shaking table tests were performed.  Figure 10 shows the shaking table and the target used in the experiments. The results of this system were compared with those measured using LVDT. 

**Figure 11:**
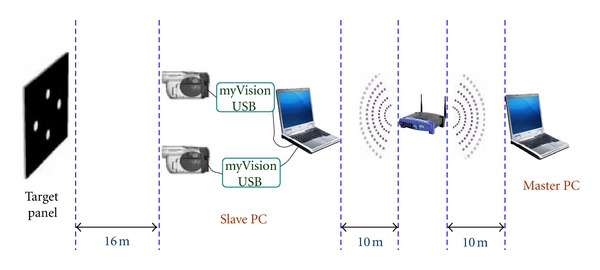
Experimental location setup.

The testing system consisted of two laptops, one Lenovo-R61 (Intel Core Duo 2.4 MHz, 2 GB of RAM), and one Acer-Asprire 5580 (Intel Core 2-Duo 1.66 MHz, 3 GB of RAM), Lenovo was used as the slave PC. Two JVC GZ-MS120 camcorders with an optical zooming capability of 40 times, a resolution of 640 × 480 pixels, and a frame rate of 30 fps were used. In addition, two *myVision USB* frame grabbers, two telescopic lenses, and one wireless LAN router complying with the 802.11 g wireless standard were implemented to transfer data between the master PC and slave PCs. The target size is 15 mm in vertical and horizontal directions. The camera was placed at 16 meters apart from the target. The number of pixels between the uppermost white spot and the lowermost white spot was 284, thus the physical resolution was 0.053 mm/pixel. The schematic of the testing configuration is given in Figure 11.

**Figure 12:**
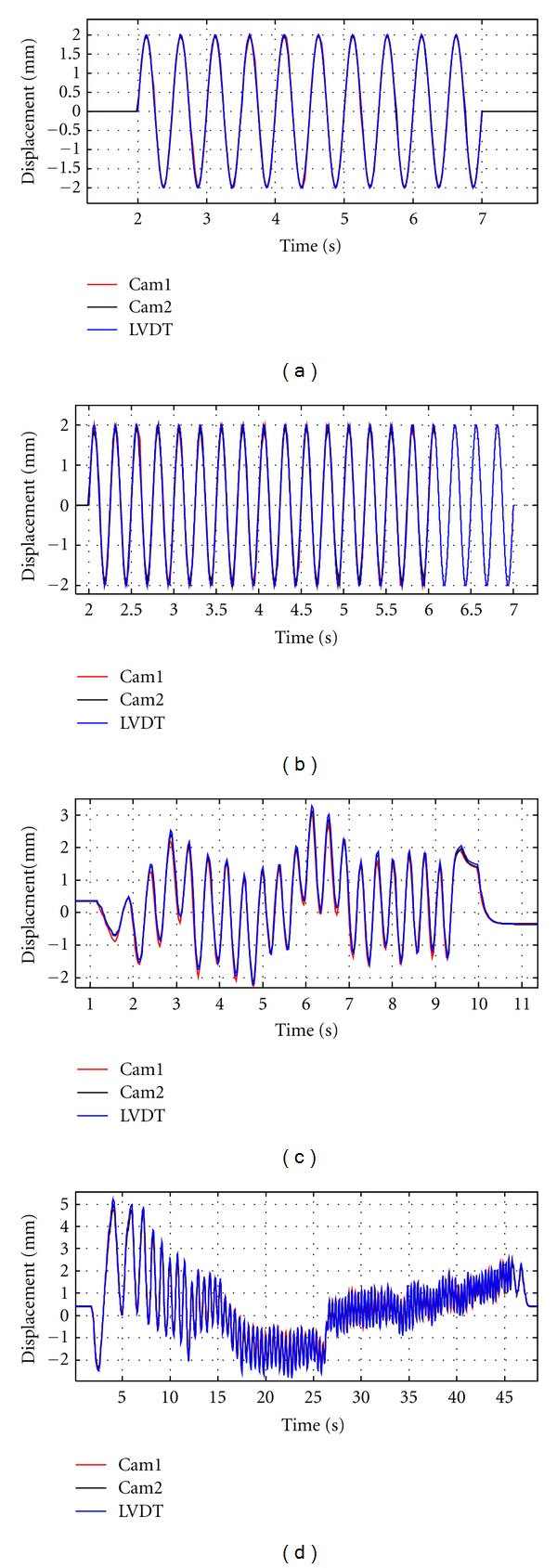
Results of shaking table test: (a) sine wave 2 Hz, (b) sine wave 4 Hz, (c) random case 1, and (d) random case 2.

Figure 12 shows the results of laboratory tests using a shaking table with 2 Hz and 4 Hz frequencies of excitation and two cases of random excitation. The outputs of this system showed very good agreement with measurements of the LVDT with the variation in the maximum values of less than 2% in all cases. 

### 3.2. Time Synchronization Test

**Figure 13:**
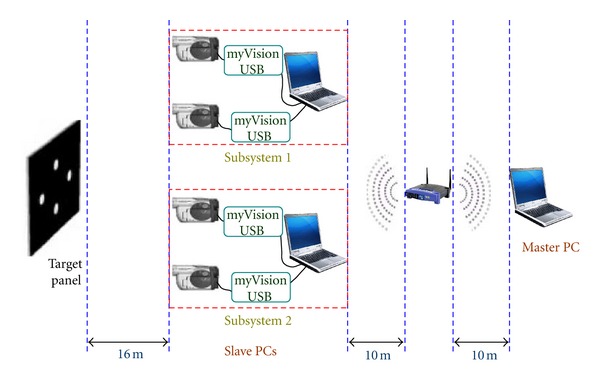
Experimental setup for the time synchronization test.

Another shaking test verified the time synchronization algorithm used in this study.  Figure 13 shows the configuration of the time synchronization test using a shaking table. In this test, three laptop PCs, one master and two slaves, were used, and the typical specifications as follows:

- Lenovo R61, Intel Core Duo 2.4 MHz, 160 of HDD, 2 GB of RAM,
- Acer Asprire 5580, Intel Core 2-Duo 1.66 MHz, 160 GB of HDD, 3 GB of RAM,
- Lenovo X201 Tablet, Intel Core i7-640LM 2.13 MHz, 320 GB of HDD, 4 GB of RAM.

Lenovo X201 Tablet was used as the master PC. Two subsystems simultaneously tracked the same target panel at a distance of 16 meters. All the subsystems were connected to master PC via a wireless LAN access point placed 10 meters away from all the PCs. The distance between the systems was determined considering the performance of telescopic lenses and camcorders. The software, including the time synchronization algorithm, was installed on both the master and the slave PCs.

**Figure 14:**
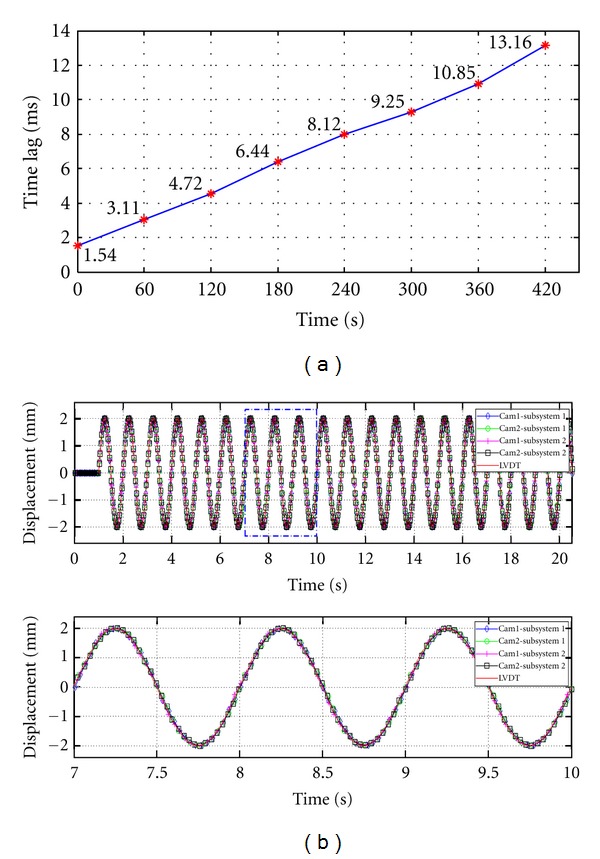
Result of the time synchronization test: (a) time lag between the subsystems and (b) displacement with an excitation frequency of 1 Hz.

The system time of the slave PCs were synchronized based on the internal time clock of the master PC. For verifying the accuracy of the salve PCs' internal time clocks, each slave PC generated voltage signals through its serial port based on its internal clock. An oscilloscope connected to the serial ports of the slave PCs monitored the voltage signals generated by the slave PCs, and the time lag between the two slave PCs was determined by referring to the voltage signals. The time lag of the subsystems were checked every 60 s and found a time delay of 1.54 ms initially after the time synchronization process. The time delay increased with time due to differences in the accuracy of the internal time clocks of the slave PCs, as shown in Figure 14(a). In this experiment, the time lag increased 1.66 ms an average of every 60 s. Thus, to avoid increasing in the time lag, the time synchronization process was performed every 60 s.  Figure 14(b) compares the displacements measured from the LVDT with those obtained from the system with the periodical time synchronization process. With sinusoidal excitation of 1 Hz, the displacements obtained from the system using different Laptop configurations were in good agreement with the LVDT measurements.

## 4. Conclusions 

In this study, an advanced synchronized multipoint vision-based system using an image processing technique has been successfully developed for a real-time dynamic displacement measurement. To evaluate the efficiency, stability, and accuracy of the system, several laboratory tests using the shaking table were conducted. Compared with the data measured from the LVDT, the outputs obtained from the proposed system showed very good agreements, and the variation in the maximum values between the LVDT measurement and system output was less than 2%.

The results of the laboratory tests showed that the proposed system could effectively be applied to large-scale civil infrastructures with low natural frequencies for real-time displacement monitoring and measurement. This system offers the following advantages over current displacement measurement systems for civil engineering.

- It simultaneously supports two camcorders at the subsystem level.
- It is easy to install, operate, and maintain.
- It can be set up quickly and configured at a low cost.
- It is robust under complicated on-site light conditions.
- It can adjust region of interest (ROI) to fit the current targets.
- It provides easy-to-expand measurement points at the subsystem level with a time synchronization process.
- It can support remote control and data transfer via an internet connection using TCP/IP.
- It can measure 2D relative motions easily and cost-effectively at two different locations using a single system supporting two cameras.
- It provides a user-friendly software interface.

In conclusion, the proposed system can be a promising and cost-effective alternative to measure displacement at multiple locations for large civil structures.
